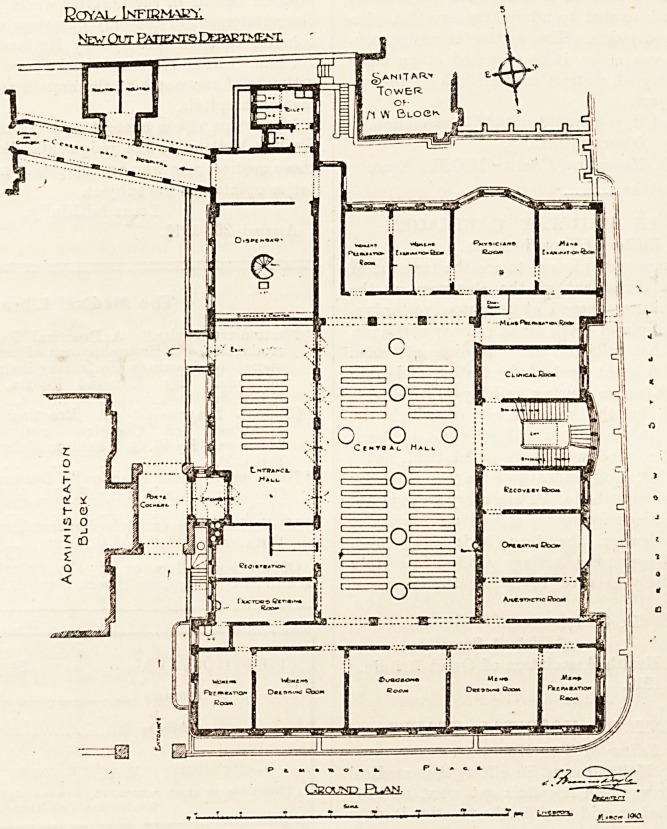# Liverpool Royal Infirmary

**Published:** 1910-09-10

**Authors:** 


### LIVERPOOL ROYAL INFIRMARY.

### NEW OUT-PATIENT DEPARTMENT.

The latest addition to this hospital occupies a site bounded on two sides by streets and on the other two by the existing hospital building. The space available was restricted, and the task of arranging the necessary accommodation could not have been an easy one. To this fact it is no doubt due that the patients enter and leave the premises by the same gate; a plan which it is important to avoid if possible.

The entrance gateway is on the north front in Pembroke Place, and the entrance door is some little distance down under a *porte-cochère*, which forms part of the original building. Passing through a lobby the patients enter a hall, in which is seating accommodation for assorted patients. This hall serves also for patients waiting for medicine.

On the right of patients as they enter is the registration office, after passing through which patients find themselves in the main waiting-hall, where seating accommodation is provided for some 200 patients.

### The Ground Floor.

The ground floor is occupied by the surgical and medical departments, the rooms for special departments being on the floor above. The medical rooms are on the south and west sides, and comprise a consulting-room, with preparation- and examination-rooms for each sex, and a clinical room.

### The Surgeon's Rooms and the Dispensary.

Between the last-named room and the surgical department is the staircase to the upper floor, in the well-hole of which is a lift. The surgeons' rooms occupy the rest of the west side and the whole of the north side. They comprise a consulting-room, preparation- and dressing-rooms for each sex, an operation-room, anæsthetic-room, and recovery-room. There is a cloak room for medical staff, and w.c. and lavatory, next the registration office.

In the south-east angle is the dispensary, which is connected with the hospital by a covered way open at the sides. Off the centre of this corridor are two isolation rooms. A small sanitary tower containing two w.c.'s and a sink room is placed at the extreme south-east angle, but whether this is intended for male or female patients does not appear. It would seem from the plan that sanitary offices for one sex only is provided on each floor.

Royal Infirmary.
New Out Patients Department

First Floor Plan.

### THE FIRST FLOOR.

The first floor is occupied by the special departments, that for women is at the south end, and comprises a large examination-room divided into three compartments, a small sterilising-room, preparation-room, and recovery-room; adjoining the last is a photographic-room. A waiting-room for this department is on the west side.

The ophthalmic department is on the west and part of the north side, and includes a consulting-room, sight-testing-room, mydriatic-room, dark-room, room for special cases, and operation-room.

### THE THROAT DEPARTMENT.

The east front is occupied by the throat department. This comprises a large examination-room, waiting-room, preparation-room, operating-room, and recovery-room. Next to the last is a nurses' room, with lavatory and w.c. attached, and a w.c. and sink-room, presumably for patients. A gallery with arches opening into the central hall runs around the four sides, and affords access to all the above rooms.

### THE BASEMENT.

The basement contains the department for skin diseases. Here is a large consulting-room, with five small boxes or cubicles for examining patients, and a private examination-room, a skin preparation-room, and a skin examination-room. A set of three bathrooms is attached to this department. Rooms for massage, with waiting-rooms, and a retiring-room for the masseuse adjoin the bathroom on the north front. A consulting-room and two waiting-rooms for lock patients completes the accommodation for patients. The remainder of the basement is occupied by a laboratory and stores for the dispensary, a sterilising-room, and the heating chamber.

The plan is an admirably simple one, and ought to work well. The only criticism we have to make is the apparent absence of sanitary accommodation for the patients.

The building has been designed by Mr. J. Francis Doyle, of Liverpool.

## Figures and Tables

**Figure f1:**
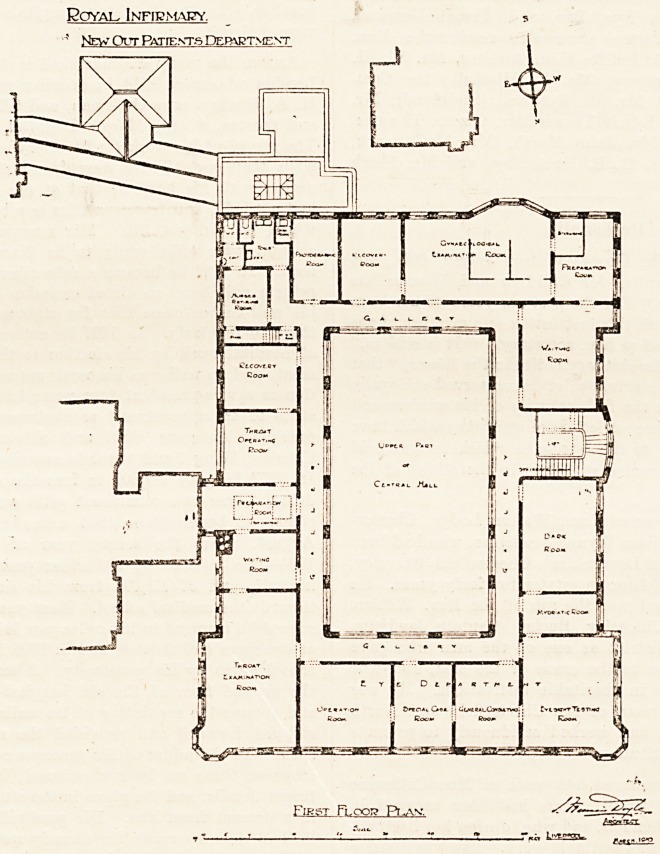


**Figure f2:**